# Home-based long-term physical endurance and inspiratory muscle training in children and adults with Fontan circulation

**DOI:** 10.3389/fcvm.2024.1411758

**Published:** 2024-09-23

**Authors:** Lena Walzer, Hannes Sallmon, Marcus Kelm, Stefan Dirks, Michael Meyer, Peter Kramer, Bernd Wolfarth, Thomas Thouet, Stanislav Ovroutski, Felix Berger, Anastasia Schleiger

**Affiliations:** ^1^ Deutsches Herzzentrum der Charité, Department of Congenital Heart Disease – Pediatric Cardiology, Berlin, Germany; ^2^ Charité – Universitätsmedizin Berlin, Corporate Member of Freie Universität Berlin and Humboldt-Universität zu Berlin, Berlin, Germany; ^3^ Department of Pediatric Cardiology, University of Graz, Graz, Austria; ^4^ German Center for Cardiovascular Research (DZHK), Berlin, Germany; ^5^ Institute of Computer-Assisted Cardiovascular Medicine, Charité – Universitätsmedizin Berlin, Berlin, Germany; ^6^ Department of Sports Medicine, Charité – Universitätsmedizin Berlin, Berlin, Germany

**Keywords:** home-based training, physical endurance training, inspiratory muscle training, Fontan circulation, pediatric and adult Fontan patients

## Abstract

**Background and study aim:**

Regular physical activity is highly recommended for patients with Fontan hemodynamics. Our aim was to investigate the effects of a long-term individualized home-based endurance training (IHET) on a bicycle ergometer in combination with inspiratory muscle training (IMT) in pediatric and adult patients after Fontan palliation. Additionally, factors influencing the trainability of Fontan palliated patients were analyzed.

**Methods:**

From 2018 to 2021 a single-center prospective study was performed initially including 25 Fontan palliated patients. During study period nine patients were excluded due to incompliance. A Magbike® bicycle ergometer (DKN Technology, France) was used for IHET and a POWERbreathe® Medic plus device (HaB GmbH, Germany) was utilized for the IMT. Over the study period, bike training was increased from 90 min of basic endurance training per week to additional 25 min of interval training per week. IMT consisted of 30 breaths per day for 6–7 days per week with pressure adaption over time. Patients underwent cardiopulmonary exercise testing (CPET) and body plethysmography including measurement of respiratory muscle strength at baseline and at follow-up examinations at 4, 10 and 22 months.

**Results:**

Follow-up examinations were completed by 18/25 patients (72.0%) at 4 and 10 months and 16/25 patients (64.0%) at 22 months. Median exercise capacity slightly increased by 0.13 W/kg from baseline to last follow-up (*p* = 0.055, 95%CI: 0.0–0.36). However, a significant increase of oxygen pulse of 0.7 ml/beat (*p* = 0.006, 95%CI: 0.38–2.22) was detectable. IMT significantly improved respiratory function with an increase of inspiratory vital capacity (VCin/reference) by 4.0% (*p* = 0.016, 95%CI: 0.8–8). Median maximal inspiratory pressure increased by 1.2 kPa (*p* = 0.003, 95%CI: 0.64–3.19) and expiratory pressure by 1.5 kPa (*p* = 0.036, 95%CI: 0.08–2.29). No adverse events or unplanned interventions occurred during the study. Patients' subjective quality of life did not significantly change over the study period.

**Conclusion:**

In Fontan palliated patients, IHET in combination with IMT leads to a significant increase in oxygen pulse, inspiratory vital capacity as well as median maximal inspiratory and expiratory pressure but not to significant improvement of quality of life. Fontan patients should be encouraged to perform regular home-based exercise training.

## 1 Introduction

The constant improvement of surgical techniques, perioperative management as well as interventional treatment strategies significantly improved long-term survival of Fontan palliated patients. Nevertheless, the unphysiological Fontan circulation and its associated complications are omnipresent and a life-limiting factor (1). The hemodynamic hallmarks of a Fontan circulation comprise a non-pulsatile pulmonary blood flow, a chronically elevated systemic venous pressure as well as a decreased ventricular pre-load resulting in reduced stroke volume and a reduced heart rate reserve during exercise (2). Furthermore, cyanosis, the lack of pulsatile flow, and the missing periods of high flow and low pressure negatively affect vessel recruitment and result in an abnormal pulmonary vasculature and increasing pulmonary vascular resistance (2). Hence, Fontan palliated patients are characterized by a reduced exercise capacity with a progressive decline starting at early adolescence (3). These physical limitations have a negative impact on quality of life (4), which is in general lower in Fontan patients compared to healthy individuals (5).

Several studies investigated the effect of resistance and endurance training in pediatric and adult Fontan patients. Short-term training programs (8–20 weeks) showed an improvement of cardiac filling, stroke volume, vital capacity as well as submaximal and peak exercise capacity and quality of life (6–10). Although current guidelines recommend incorporating physical exercise programs as well as inspiratory muscle training in Fontan follow-up-care (11, 12), the availability and feasibility of exercise programs, especially in rural areas is limited. Consequently, home-based exercise training was suggested as a promising alternative (8, 10).

In this study, we investigated the beneficial effects of an individually adapted home-based endurance training on a bicycle ergometer in combination with inspiratory muscle training in pediatric and adult patients with Fontan circulation. Another important study aim was to analyze the trainability of Fontan palliated patients and to determine which factors influence the improvement of fitness in this patient cohort. Preliminary results after 10 months of follow-up have previously been published (13); in this manuscript we specifically report the results after 22 months of follow-up.

## 2 Material and methods

### 2.1 Study design

In this single-center prospective study we aimed to examine the effects of an individually adapted home-based physical endurance training (IHET) combined with inspiratory muscle training (IMT) on patients with Fontan circulation. Primary study endpoints were cardiopulmonary exercise testing (CPET) parameters such as maximal oxygen consumption (VO_2_max), heart rate reserve or relative power as well as maximal inspiratory or expiratory pressure (MIP/MEP), total lung capacity (TLC) or inspiratory vital capacity (VC_insp_). Secondary study endpoints were the occurrence of adverse events. Patients followed routinely at our outpatient clinic were enrolled between March 2018 and March 2021. The study consisted of two phases. Phase I involved follow-up examinations after 4 and 10 months, the preliminary results of Phase I were previously published (13). Phase II included one follow-up visit at study end after 22 months of training. Inclusion criteria consisted of a minimum patient age of 6 years, a minimum height of 120 cm and the compliance to perform IHET and IMT throughout the whole study period. Exclusion criteria were defined as adverse events during baseline CPET, such as syncope, severe oxygen desaturation, symptomatic hypotonia or arrhythmias. Out of the 25 patients who were initially recruited, 9 patients were excluded due to incompliance. None of the pre-screened patients were excluded due to adverse events during baseline CPET. Informed consent of patients or legal guardians was obtained before enrollment. The study was approved by the institutional review board and the ethics committee of Charité—Universitätsmedizin Berlin (decision number: EA2/244/17). In addition to IHET, patients completed a standardized health-related quality of life questionnaire (PedsQL^TM^–Pediatric Quality of Life Inventory) including the cardiac module (14). Systolic ventricular function was measured by echocardiography based on the modified Simpson's method (15). Atrioventricular valve incompetence (AVVI) was classified as absent/mild, moderate, or severe by visual assessment of the regurgitation jet dimensions in color Doppler sonography. We used the following reference values to evaluate the obtained measurements: data from the German Health Interview and Examination Survey for Children and Adolescents (KiGGs) for pediatric body weight (16) and global lung function initiative (GLI) reference values (17) for pulmonary function.

### 2.2 Training protocol

The home-based training program was previously described in detail by our study group (13). Briefly, IHET consisted of the combination of basic endurance and interval training, which was individually adapted based on baseline CPET values. Endurance training workload was set as 55% of the maximal workload obtained during baseline CPET, whereas during interval training 80% were obligatory. Participants performed 90 min of basic endurance training per week during the first 4 months, which was extended by 25 min of interval training after the first follow-up visit. Additionally, the training program was adjusted after each follow-up CPET according to the individual performance. Inspiratory muscle training was performed six to seven times a week with an effort-adjusted resistance. The training protocol was developed in cooperation with the Department of Sports Medicine at Charité—Universitätsmedizin Berlin. The Magbike® AM-5i/3i (DKN Technology®, Clermont-Ferrand, France) cycle ergometer was used for the endurance training. The DKN AM 5i/3i Magbike® is a high intensity ergometer, which provides a high number of training programs and various resistance levels enabling users to adjust intensity of workout. Every participant was supplied with an activity tracker (Fit Bit Inc®, San Francisco, California). IMT was performed using a handheld “POWERbreathe®Medic plus” device (POWERbreathe®, Winsen, Germany) with adjustable expiratory resistance. During the study period, patients, parents or legal guardians filled out a training log, which was submitted to our institution once a month. Compliance was monitored by regular phone calls.

### 2.3 Statistical analysis

Data were collected from medical records of the German Heart Center of the Charité. CPET and IMT data are expressed as median and interquartile range and were obtained at the same sequence of events at baseline and after 4, 10 and 22 months of follow-up. Data were examined using a response profile analysis which characterizes the change of means over time (18). A mixed effects model with unstructured covariance was used for the analysis of the longitudinal CPET and IMT parameters from baseline and subsequent follow-up visits. This model estimates the change of means from baseline to the predefined time points. The area under the curve minus baseline (AUC) was used supplemental to the analysis of single time points to provide an overall assessment of the training effect. AUC was calculated using linear combinations of the coefficients to estimate an overall effect from baseline to month 22 (18). A positive AUC value describes an overall increase of the corresponding variable from baseline to follow-up end, a negative AUC corresponds with a decrease of a parameter over time. *P*-values <0.05 were considered statistically significant. Statistical analysis was performed using Stata software version 18.0 (Stata Corp, College Station, Tx, USA), graphs were created using GraphPad Prism version 10.1.2. for Windows (GraphPad Software, Boston, Massachusetts, USA).

## 3 Results

### 3.1 Patient characteristics

Out of 25 patients initially included in the study, follow-up examinations were completed by 18/25 patients (72.0%) at 10 months and 16/25 patients (64.0%) at 22 months. In the following, patient characteristics of the study completers are described. Median patient age was 15.0 years [12.0; 23.5]. Seven patients were male, nine patients were female. Median age at Fontan palliation was 3.2 years [2.5; 6.2]. Thirteen patients had a systemic left ventricle. Tricuspid atresia was the most common underlying morphology (Table 1). Fontan modifications included extra-cardiac conduit in 13/16 (81%) patients, lateral tunnel in 2/16 patients (13%) and atrio-pulmonary connection in 1/16 patients (6%). In four patients, the fenestration was patent at baseline examination. In this subcohort, median oxygen saturation at rest was 95.0% [92.0; 95.0] and decreased to 87.0% [85.0; 87.0] during exercise. Two patients had received a pacemaker due to sick sinus syndrome or complete AV block before study initiation. Echocardiographically determined systolic function of the systemic ventricle was normal in eleven patients and mildly impaired in five patients. Atrioventricular valve regurgitation was absent/mild in fifteen patients and moderate in one patient. Fourteen out of sixteen patients underwent cardiac catheterization 1.3 years [0.1; 2.0] before or 0.8 years [0.2; 2,6] after study initiation. Invasively measured median pulmonary artery pressure was 11.0 [10.0; 13.5] mmHg, median transpulmonary gradient 3.5 [3.0; 4.0] mmHg, and end-diastolic systemic ventricular pressure 10.0 [7.0; 12.5] mmHg. During the study period the following interventions were performed: closure of venovenous collaterals (*n* = 3), implantation of a stent into the left pulmonary artery (*n* = 1), implantation of a stent into the Fontan conduit (*n* = 2), closure of an arteriovenous collateral (*n* = 1) and balloon dilatation of the Fontan conduit (*n* = 1). Medical heart failure treatment included ACE inhibitors in 8/16 patients (50%), beta blockers in 1/16 patients (6%), and diuretics in 5/16 patients (31%). One patient received antiarrhythmics for treatment of intermittent atrial flutter. This medication remained unchanged during the study period.

**Table 1:** Patient characteristics of study completers.

Parameter	Median [IQR] or frequency (%)
Patient age (years)	15.0 [12.0; 23.5]
Age at Fontan operation (years)	3.2 [2.5; 6.2]
Gender (male)	7/16 (44%)
Patient weight (kg)	38.5 [47.5; 61.5]
Body mass index (kg/m²)	31.4 [25.4; 36.7]
Cardiac malformation	
Tricuspid atresia	9/16 (56%)
Pulmonary atresia with ventricular septal defect	2/16 (13%)
Double-inlet left ventricle	2/16 (13%)
Hypoplastic left heart syndrome	1/16 (6%)
Mitral atresia	1/16 (6%)
Double-outlet right ventricle	1/16 (6%)
Left systemic ventricle	13/16 (81%)
Fontan type	
Extra-cardiac conduit	13/16 (81%)
Lateral tunnel	2/16 (13%)
Atrio-pulmonary Connection	1/16 (6%)
Patent fenestration	4/16 (25%)
Transcutaneous saturation at rest (%)	97 [95; 98]
Transcutaneous saturation during exercise (%)	92 [89; 95]
Medical treatment	
ACE inhibitors	8/16 (50%)
Beta blockers	1/16 (6%)
Diuretics	5/16 (31%)
Impairment of systolic ventricular function	
None	11/16 (67%)
Mild	5/16 (31%)
Atrioventricular valve regurgitation	
absent/mild	15/16 (94%)
Moderate	1/16 (6%)
Invasive hemodynamic evaluation	
mPAP* (mmHg)	11.0 [10.0; 13.5]
TPG* (mmHg)	3.5 [3.0; 4.0]
EDP** (mmHg)	10.0 [7.0; 12.5]

Data of study completers (*n* = 16) are presented as median and [interquartile range] or frequency (percent). ACE, Angiotensin converting enzyme; mPAP, Median pulmonary artery pressure; IQR, interquartile range, TPG, Transpulmonary pressure gradient.

*n = 14/16 patients. **n = 8/16 patients.

### 3.2 Cardiopulmonary exercise capacity testing

**Figure 1:**
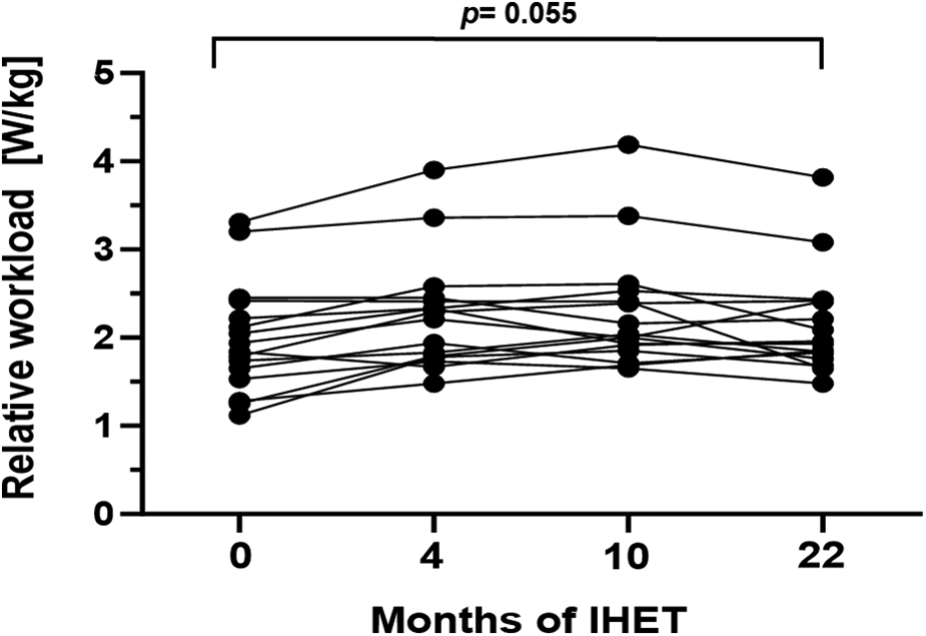
Maximum relative workload (W/kg) during the 22-month endurance training period. Values are provided at baseline, after 4, 10 and 22 months of follow-up. Each line represents one patient (*n* = 16).

Exercise capacity was evaluated via CPET at baseline and at every scheduled follow-up examination at 4, 10, and 22 months, respectively (Table 2). During the study period, median exercise capacity increased by 0.13 W/kg (*p* = 0.055, 95%CI: 0.00–0.36; Figure 1) after 22 months. The area under the curve suggests an overall increase of exercise capacity from baseline to the last follow-up examination (*p* = 0.004, Table 2). Oxygen pulse increased significantly by 0.7 ml/beat (*p* = 0.006, 95%CI: 0.38–2.22). There was no statistically significant increase in peak oxygen uptake or VE/VCO_2_-slope values over the 22-month training period (Table 2). Furthermore, no significant changes in resting heart rate, maximum heart rate or heart rate reserve were detected (Table 2). Transcutaneous oxygen saturation at rest significantly decreased by 1.0% (*p* = 0.009; 95%CI: −1.82 to −0.26) over the study period. Accordingly, transcutaneous oxygen saturation during exercise significantly decreased by 2.0% (*p* < 0.001*,* 95%CI: −2.31 to −0.89, Table 2).

**Table 2:** Cardiopulmonary exercise testing and respiratory function.

	Baseline (*n* = 18)	4-month FU (*n* = 18)	10-month FU (*n* = 18)	22-month FU (*n* = 16)	95%CI	*P*-Value	AUC	AUC *P*-Value
Heart rate at rest (bpm)	89 [82; 97]	97 [87; 104]	86 [77; 94]	89 [79; 104]	−8.6 to 4.3	0.52	−26.1	0.523
Heart rate during exercise (bpm)	150 [123; 155]	152 [127; 166]	149 [134; 162]	150 [133; 169]	−5.7 to 10.4	0.562	62.3	0.386
Heart rate reserve (bpm)	82 [34; 95]	71 [58; 91]	82 [65; 90]	79 [68; 92]	−4.8 to 13.8	0.341	89.2	0.263
TCS at rest (%)	97 [95; 98]	96 [94; 98]	96 [94; 98]	96 [95; 97]	−1.82 to −0.26	**0**.**009**	−16.3	**0**.**02**
TCS during exercise (%)	94 [93; 95]	94 [91; 95]	92 [90; 95]	92 [89; 95]	−2.31 to −0.89	**<0**.**001**	−23.1	**0**.**007**
Max. power/weight (W/kg)	1.8 [1.4; 2.2]	2.1 [1.7; 2.4]	2.0 [1.7; 2.4]	1.9 [1.8; 2.4]	0.00 to 0.36	0.055	4.4	**0**.**004**
VO_2_max (ml/kg/min)	24.6 [19.5; 29.8]	24.9 [20.6; 30.9]	27.2 [22.9; 31.7]	25.7 [23.8; 29.5]	−1.42 to 4.32	0.323	26.2	0.232
O_2_ pulse (ml/beat)	7.5 [6.5; 10.6]	7.8 [6.2; 10.5]	8.3 [7.5; 9.9]	8.2 [7.6; 9.9]	0.38 to 2.22	**0**.**006**	16.3	**0**.**006**
VE/VCO_2_ slope	32.6 [28.0; 34.7]	33.7 [30.3; 34.8]	33.0 [26.9; 35.3]	31.9 [28.3; 35.4]	−2.29 to 3.61	0.661	7.3	0.794
FEV_1_ (l)	2.5 [2.0; 3.4]	2.6 [2.1; 3.3]	2.6 [2.1; 3.3]	2.6 [2.3; 3.3]	0.06 to 0.31	**0**.**003**	1.3	0.092
VC_insp_ (l)	2.6 [2.1; 3.5]	2.6 [2.3; 3.5]	2.9 [2.3; 3.5]	3.0 [2.6; 3.6]	0.15 to 0.47	**<0**.**001**	3.0	**0**.**02**
FEV_1_/VC_in_	0.94 [0.87; 0.97]	0.91 [0.85; 0.97]	0.92 [0.82; 0.95]	0.9 [0.86; 0.93]	−0.08 to 0.00	**0**.**027**	−0.5	0.104
VC insp/reference (%)	79 [72; 92]	85 [69; 93]	81 [75; 89]	83 [72; 94]	0.8 to 7.97	**0**.**016**	25.5.	0.47
TLC/reference (%)	84 [73; 92]	85 [77; 94]	84 [76; 91]	82 [78; 96]	−4.27 to 2.14	0.515	−7.4	0.819
FEV1/VC insp (%)	94 [87; 97]	91 [86; 97]	93 [83; 96]	90 [86; 93]	−7.82 to −0.45	**0**.**028**	−50.1	0.167
MIP (kPA)	6.5 [4.8; 8.6]	8.9 [7.1; 11.0]	8.3 [6.5; 10.2]	7.7 [6.1; 11.1]	0.64 to 3.19	**0**.**003**	44.6	**<0**.**001**
MEP (kPA)	5.9 [5.4; 8.4]	8.5 [4.8; 9.6]	7.6 [5.9; 10.1]	7.4 [5.7; 10.5]	0.08 to 2.29	**0**.**036**	22.4	**0**.**004**
BORG scale	13 [12; 14]	14 [13; 15]	14 [13;15]	14 [13; 15]	−0.2 to 1.9	0.126	17.1	**0**.**034**

Data are presented as median and [interquartile range]. 95% CI refers to the change from baseline to month 22. AUC, area under the curve; BMI, body-mass-index; FEV_1_, forced expiratory volume in one second; FU, follow-up, MEP, maximal expiratory pressure; MIP, maximal inspiratory pressure; TCS, transcutaneous saturation; TLC, total lung capacity; VC_insp_, inspiratory vital capacity; VO_2_max, maximal oxygen consumption; VE/VCO_2_, minute ventilation/carbon dioxide production. Baseline, 4- and 10-month-FU: *n* = 18; 22-month FU: *n* = 16. *P*-value refers to the selective change from baseline to 22-months follow-up. AUC *P*-value refers to the change over time from baseline to follow-up end.

Statistically significant values are highlighted in bold.

### 3.3 Respiratory function

**Figure 2:**
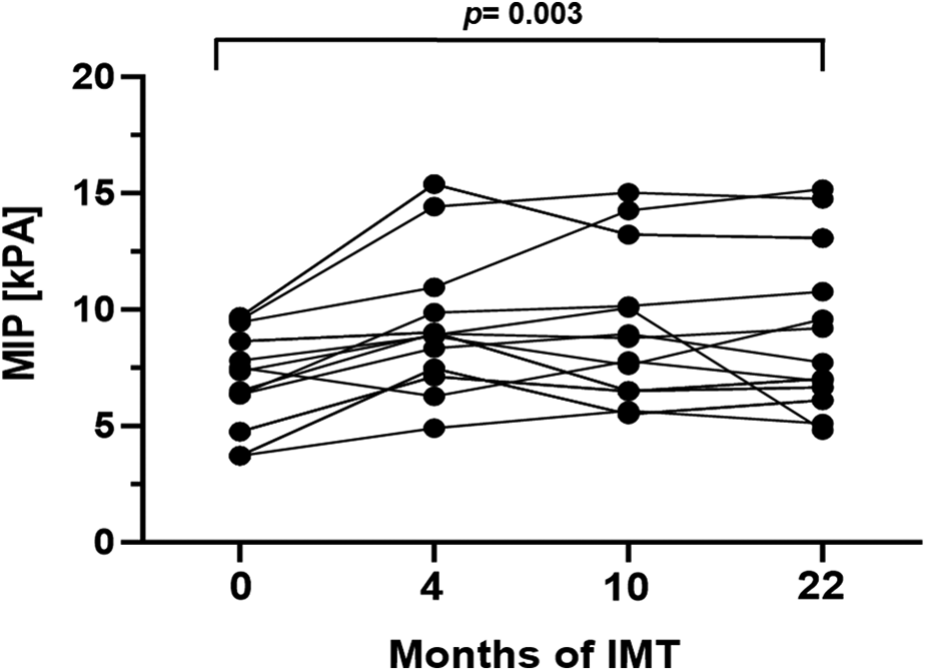
Mean inspiratory pressure (kPa) during the 22-month inspiratory muscle training period. Values are provided at baseline, after 4, 10 and 22 months of follow-up. Each line represents one patient (*n* = 13).

**Figure 3:**
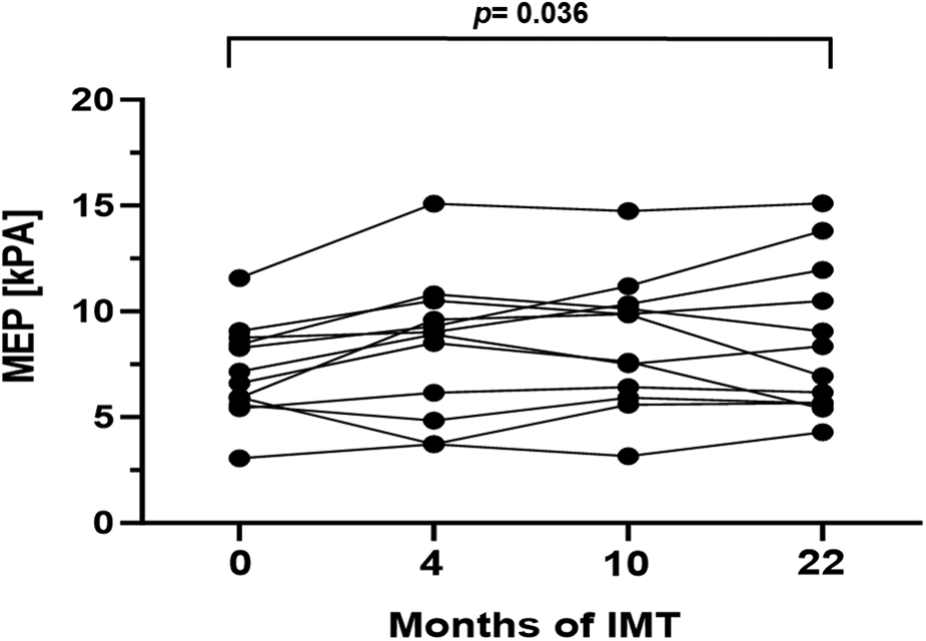
Mean expiratory pressure (kPa) during the 22-month inspiratory muscle training period. Values are provided at baseline, after 4, 10 and 22 months of follow-up. Each line represents one patient (*n* = 13).

In order to evaluate respiratory function, body plethysmography and measurement of respiratory muscle strength were performed at baseline and follow-up examinations. Prior to study initiation lung function was normal in 10/16 patients (63%), while 2/16 patients (13%) presented an obstructive pattern and 4/16 patients (25%) a restrictive pattern. After 22 months of training, age- and gender adjusted inspiratory vital capacity increased significantly by 4.0% (*p* = 0.016, 95%CI: 0.8–7.97). Additionally, a significant increase of median MIP and MEP was detected (Figures 2, 3). The Tiffeneau-Pinelli index (FEV_1_/VC) significantly decreased by 4% (*p* = 0.028, 95%CI: −7.82 to −0.45). Total lung capacity showed no significant change over the study period.

### 3.4 Potential factors influencing the trainability of Fontan palliated patients

Trainability was defined as a significant change of the parameter relative power (W/kg) from baseline to follow-up end. The median overall improvement of relative power in the total cohort was 0.1 W/kg (*p* < 0.001; 95%CI: 0.12–0.32) over time. The increase of relative power was by 0.1 W/kg (*p* = 0.049; 95%CI: −0.38 to 0.0) higher in patients classified with NYHA class I compared to patients classified with NYHA class II. There was no detectable influence of age, gender, systolic ventricular function, or the absence of sinus rhythm on the trainability of Fontan palliated patients (Supplementary Table 1).

### 3.5 Differences between study completers and drop-outs

No statistically significant differences between study completers and dropouts concerning age, sex, diagnosis, body dimensions or NYHA class were detected (Supplementary Table 2). The number of patients receiving beta blockers was significantly higher in the dropout group (4/9, 44%) compared with the completer group (1/16, 6%, *p* = 0.04).

### 3.6 Quality of life

Patients' subjective quality of life as assessed by the Peds QL™ 4.0 and the Peds QL™ 3.0 cardiac module did not show any significant changes after 22 months of training (Table 3). As indicated by the AUC, there is some evidence for an overall benefit in the subcategory Heart Problems and Treatment from baseline to month 22 over time (*p* = 0.019). No significant changes in the other subcategories were detected (Table 3). The average Psychosocial Health Summary Score and the Physical Health Summary Score also did not significantly differ between baseline and last follow-up (Table 3).

**Table 3:** Assessment of quality of life.

	Baseline	4-month FU	10-month FU	22-month FU	95%CI	*P*-value	AUC	AUC *P*-value
Peds QL™ 4.0								
Total Score	74 [67; 80]	77 [72; 83]	75 [68; 88]	76 [67; 87]	−4.0 to 8.7	0.464	56.5	0.257
Psychosocial Health Summary Score	72 [63; 78]	75 [68; 80]	69 [63; 88]	75 [65; 87]	−4.8 to 10.7	0.462	58.6	0.305
Physical Health Summary Score	83 [73; 92]	84 [75; 94]	89 [77; 95]	86 [73; 91]	−4.6 to 6.1	0.782	50.3	0.305
Peds QL™ 3.0 cardiac module								
Heart Problems and Treatment	68 [55; 75]	68 [64; 79]	68 [64; 82]	71 [61; 89]	−2.3 to 12.8	0.170	**117**.**9**	**0**.**019**
Treatment II	93 [90; 98]	90 [85; 100]	90 [88; 98]	92 [80; 95]	−7.3 to 5.9	0.834	10.9	0.851
Perceived Physical Appearance	75 [67; 88]	75 [54; 91]	79 [67; 100]	79 [54; 96]	−10.2 to 6.0	0.614	16.7	0.81
Treatment Anxiety	88 [66; 97]	85 [69; 97]	81 [69; 97]	81 [75; 94]	−6.0 to 4.7	0.813	−41.4	0.625
Cognitive Problems	68 [48; 73]	70 [48; 75]	67 [55; 88]	67 [53; 88]	−0.9 to 15.0	0.084	84.6	0.239
Communication Problems	71 [54; 83]	67 [63; 79]	67 [54; 96)	71 [54; 92]	−1.4 to 13.9	0.110	92.2	0.313

Data are presented as median and [interquartile range], (*n* = 16). 95% CI refers to the change from baseline to month 22. AUC, Area under the curve. *P*-value refers to the selective change from baseline to 22-months follow-up. AUC *P*-value refers to the change over time from baseline to follow-up end.

Statistically significant values are highlighted in bold.

### 3.7 Adverse events

No adverse events or unplanned interventions occurred during the study.

## 4 Discussion

In this study we investigated the effect of an individualized home-based endurance training combined with inspiratory muscle training in patients after Fontan palliation over a period of 22 months. Our data reveal a positive impact of IHET on oxygen pulse and vital capacity as variables of cardiopulmonary exercise capacity as well as maximal expiratory and inspiratory pressure as measures of respiratory muscle strength. Nonetheless, these beneficial physical changes were not accompanied with a subjective improvement of quality of life.

As the population of Fontan palliated patients is predicted to substantially grow over the next 30 years, the aspect of long-term morbidity needs further attention (19). It has been demonstrated that several cardiopulmonary exercise testing parameters are associated with increased morbidity and mortality (20). On this account, cardiopulmonary exercise testing is recommended as a regular follow-up examination for patients with Fontan circulation (21). In general, exercise capacity has been shown to be significantly reduced in Fontan patients, with a start of decline as early as adolescence (3, 22). Nevertheless, multiple studies have shown the safety as well as positive effects of regular physical activity on exercise capacity, cardiac function, and quality of life (7, 23). Most previously published studies on endurance training in Fontan patients reported a rather short training period (3–12 months (24). To our knowledge this is the first study describing the effects of an exercise intervention over a period of 22 month.

### 4.1 Cardiopulmonary function

In Fontan palliated patients, limited exercise capacity is caused by a combination of multiple cardiovascular, respiratory, musculoskeletal, neurodevelopmental, and psychosocial factors (25). Missing a subpulmonary ventricle, the Fontan patients' ability to increase their preload to improve cardiac output is limited (26). Therefore, heart rate reserve determines exercise capacity, but is often impaired by congenital or postoperative sinus node dysfunction or medication (27). While the average exercise capacity has been reported to be reduced to about 60% (28, 29), oxygen pulse—a surrogate of stroke volume—was identified as the primary cardiac determinant, accounting for 73% of predicted peak oxygen consumption (VO_2_max) (30). In our investigation, 22 months of exercise training did not result in a significant improvement in resting heart rate or heart rate reserve. In our 10-month follow-up report on the same cohort, it was hypothesized that subtle changes in heart rate reserve might become evident with a longer training period (13), which we could not validate after 22 months. Since our cohort only included two patients with a pacemaker as well as two patients on antiarrhythmic medication/beta blockers, sub-analyses of these subgroups were not possible. Unfortunately, we could not uncover whether intrinsic factors in all Fontan patients or individual factors like non-sinus-heart-rhythm or antiarrhythmic medication are responsible for missing improvement of the heart rate reserve.

When compared to healthy individuals, Fontan patients showed a significantly lower peak oxygen pulse, which is a valid predictor of stroke volume during exercise (31). Remarkably, we revealed a significant improvement of oxygen pulse over the training period, indicating an increase in stroke volume through workout.

When analyzing exercise capacity, VO_2_max is probably the most referred-to value. In the previously published 10-month follow-up, an increase of VO_2_max was shown for the subgroup-analysis of adult/teenage patients (13). We were unable to reproduce these results for the 22-month follow-up. A recent review on exercise studies in Fontan patients pointed out that only 9/16 studies (56%) could show a significant change in VO_2_max (24). The lack of a statistically significant improvement of VO_2_max over the study period might additionally be explained by the high baseline VO_2_max values of our cohort, which might not be altered significantly by an exercise training intervention. The incapability of Fontan patients to enhance stroke volume and cardiac output may reach a plateau and reduce the potential increase in VO_2_max (26). Furthermore, several studies have shown a progressive decline in VO_2_max for Fontan patients starting in adolescence (3, 30) with values ranging from 0.8 ± 1.7 (28) over 1.3 ± 0.4 (29) to 2.6 ± 2.7%/year (22). Therefore, our results of a non-significant but steady increase of VO_2_max over the study period of 22 months could be interpreted as a success, since without endurance training a decline would have been expected. This finding emphasizes the potential of an individualized long-term exercise intervention to delay hemodynamic deterioration and the inevitable failure of the Fontan circulation.

Oxygen saturation remains slightly reduced after Fontan palliation. Possible causes could be intrapulmonary right-to-left shunting, venovenous collaterals, persistent fenestration, and coronary sinus blood return to the pulmonary venous atrium (32). Moreover, it was described that oxygen saturation decreases even further with exercise (20, 29, 33). In our cohort, after 22 months of training oxygen saturation at rest and maximum capacity decreased slightly but significantly. These findings are consistent with a previous study describing a significant decrease in oxygen saturation at peak performance with follow-up duration after Fontan palliation (33).

### 4.2 Pulmonary function

A restrictive ventilatory pattern has been previously described in Fontan patients, possibly caused by diaphragmatic paralysis, scoliosis, a high total number of thoracotomies, and low body mass index (4, 9). In accordance with this, 25% of the participants of our study showed a restrictive lung function at baseline. Studies analyzing the impact of exercise training and/or inspiratory muscle training on lung function have yielded variable results. We were able to show an increase of relative inspiratory vital capacity matching the results of a previous study demonstrating an increase in vital capacity after a 12-week endurance training program (9). Our results show a significant decrease of the Tiffeneau-Pinelli-Index (FEV_1_/VC_in_), which is explained by a greater increase of the parameter VC_in_ than FEV_1_ over the study period. As restrictive lung physiology is dominant in Fontan patients, we would consider the greater increase of the inspiratory vital capacity a positive result. More importantly, our results display a significant improvement of median MIP and MEP, which corresponds with findings of other investigators (30, 32). Since improved inspiratory strength is associated with an increased pulmonary blood flow and cardiac output (34), these findings emphasize the importance of respiratory training in Fontan palliated patients.

### 4.3 Possible influences on trainability of Fontan palliated patients

Our findings revealed no association between the increase of overall relative power with age, follow-up duration since Fontan operation, gender, and body mass index. In our cohort the increase of relative power was lower in patients classified with NYHA functional class II compared to patients with NYHA class I, suggesting that endurance training is more beneficial if initiated before the onset of clinical deterioration. Studies analyzing the influence of baseline characteristics such as imaging results, medical treatment, hemodynamic parameters measured during catheterization or laboratory parameters on exercise capacity in Fontan patients are rare. A recent study showed an influence of mean pulmonary artery pressure and transpulmonary pressure gradient on peak oxygen uptake (35). We were unable to detect an effect of patient characteristics on the change of physical capacity from baseline, which might be explained by the small sample size and the heterogeneous cohort. Additionally, invasively measured hemodynamic parameters were not available for all participants and subgroups presenting specific baseline characteristics were rather small. For example, only 2/16 (12.5%) study completer were on antiarrhythmic drugs/beta blockers.

### 4.4 Health-related quality of life

We could not detect a significant improvement in patients' subjective health-related quality of life after 22 months of training (except for the subcategory Heart Problems and Treatment). Previous studies on the impact of exercise training on self-reported quality of life showed inconsistent results: While a few studies detected a positive impact of physical training on quality of life (6, 8), these findings could not be confirmed by other investigators (36) or our study. Additionally, in studies showing a positive effect, physical activity was reported to be beneficial only in a specific age group (37), or in the parents' but not the patients' perception (38). These differences could be explained by varying training programs or methods as well as varying supervision or motivation by study staff. In addition, self-reported quality of life can be cofounded by many other factors, such as illusory expectations, peer comparison, parental influences, or inattentiveness during questionnaire completion. Aside from this, some analyzed subcategories of the questionnaire, such as treatment anxiety or cognitive problems and communications, are unlikely to be influenced by physical exercise training. It also has to be considered that the baseline scores in our analysis were already high. As mentioned in our 10-month-follow up report (13), this study was carried out while the entire world was heavily affected by the SARS-CoV-2 pandemic. Closed schools, mandatory working from home, reduced social contacts, and limited activities had a huge impact on everybody's quality of life. As patients with congenital heart defects are considered a high-risk group, they often reduced their social life and interactions even more in fear of infection.

## 5 Limitations

There are several limitations to this study. This study was severely influenced by the occurrence of the SARS-CoV-2 pandemic. Due to governmental restrictions at specific times during the study period we were not always able to conduct CPET. Consequently, the study did not comprise four follow-up examinations as initially planned but only three. The small number and heterogeneity of the study cohort as well as the lack of an age and gender matched control group might distort the statistical analysis of this study. Since our study did not include a body composition assessment, further investigations are required to provide more detailed information concerning IHET induced changes of skeletal muscle or fat mass. Another limiting factor are the differences in parental surveillance and support during the home-based training, as well as differences in cognitive abilities and intrinsic motivation, ambition, and discipline. Lastly, learning effects have to be mentioned. Finally, although the PedsQL™ questionnaire was originally designed for pediatric patients, in this study it was used in adult patients to provide a comparable quality of life analysis.

## 6 Conclusion

We demonstrate that individualized, home-based endurance training in combination with inspiratory muscle training is safe and effective for patients with Fontan circulation. Some variables of CPET as well as MIP and MEP significantly improved over the study period whereas subjective quality of life was not influenced by exercise intervention. Therefore, Fontan patients should be encouraged to perform home-based training on a regular basis.

## Data Availability

The datasets presented in this article are not readily available due to patient privacy. Requests to access the datasets should be directed to the corresponding author.
