# Supplementary material for: Home-based long-term physical endurance and inspiratory muscle training in children and adults with Fontan circulation
Source: Front Cardiovasc Med. 2024 Sep 23;11:1411758. doi: 10.3389/fcvm.2024.1411758 (PMC11456991; doi:10.3389/fcvm.2024.1411758)
Supplement: Supplementary file 1 [file Table1.pdf]

Supplementary Table 1: Potential factors influencing the trainability of Fontan palliated patients

Parameter	Estimate	SE	p-Value	95% CI
Gender	-0.113	0.051	0.271	-0.313 to 0.088
NYHA class	-0.189	0.096	**0.049**	-0.377 to – 0.001
Age (years)	-0.008	0.005	0.141	- 0.019 to 0.003
BMI (kg/m²)	-0.005	0.017	0.747	-0.038 to 0.027
Weight (kg)	-0.002	0.004	0.609	-0.009 to 0.005
Training time (min)	-0.002	0.002	0.264	-0.005 to 0.001
Ventricular function	0.158	0.114	0.164	-0.065 to 0.38
Time after Fontan operation (days)	-0.002	0.007	0.786	-0.015 to 0.012
Presence of sinus rhythm	-0.185	0.163	0.257	-0.504 to 0.135

Data were analysed using a univariate mixed effects model. Statistically significant values are highlighted in bold. BMI = body mass index, NYHA = New York Heart Association.

Supplementary Table 2: Differences between study completers and drop-outs

Parameter	Completer (n=16)	Drop-out (n=9)	P-Value
Patient age (years)	15.0 [12; 23.5]	21.0 [12.0; 30.0]	0.116
Gender (male)	7/16 (43.8 %)	5/9 (55.6)	0.42
Patient weight (kg)	47.5 [38.5; 61.5]	62.0 [50.5; 77.0]	0.16
Cardiac malformation			
• Tricuspid atresia	9/16 (56 %)	5/9 (56 %)	0.555
• Pulmonary atresia with ventricular septal defect	2/16 (13 %)	0/0 (0 %)	0.236
• Double-inlet left ventricle	2/16 (13 %)	1/9 (11 %)	0.721
	1/16 (6 %)	0/0 (0 %)	0.667

• Hypoplastic left heart syndrome<br>• Mitral atresia<br>• Double-outlet right ventricle	1/16 (6 %)<br>1/16 (6 %)	0/0 (0 %)<br>3/9 (33 %)	0.667<br>0.093
Left systemic ventricle	13/16 (81 %)	6/9 (67 %)	0.55
Medical treatment<br>• ACE inhibitors<br>• Beta blockers<br>• Diuretics	 8/16 (50 %)<br>1/16 (6 %)<br>5/16 (31 %)	 1/9 (11 %)<br>4/9 (44 %)<br>4/9 (44 %)	 0.062<br>**0.04**<br>0.407
NYHA Class<br>• I<br>• II	 9/16 (56 %)<br>9/16 (56 %)	 6/9 (67 %)<br>3/9 (33 %)	 0.47<br>0.248

Data are presented as median [interquartile range] or frequency (percent). ACE=Angiotensin converting enzyme; NYHA: New York Heart Association.
